# Association Between the Use of DPP4 Inhibitors and Metformin and the Risk of Cancer in Patients with Type 2 Diabetes: A Multicenter Retrospective Cohort Study Using the OMOP CDM Database

**DOI:** 10.3390/cancers17223620

**Published:** 2025-11-10

**Authors:** Gyu Lee Kim, Yu Hyeon Yi, Jeong Gyu Lee, Young Jin Tak, Seung Hun Lee, Young Jin Ra, Byung Kwan Choi, Sang Yeoup Lee, Young Hye Cho, Eun Ju Park, Youngin Lee, Jung In Choi, Sae Rom Lee, Ryuk Jun Kwon, Soo Min Son

**Affiliations:** 1 Department of Family Medicine and Medical Research Institute, Pusan National University Hospital, Busan 49241, Republic of Korea; happygaru@hanmail.net (G.L.K.); eltidine@hanmail.net (J.G.L.); 03141998@hanmail.net (Y.J.T.); greatseunghun@hanmail.net (S.H.L.); yjra80@naver.com (Y.J.R.); 2 Department of Family Medicine, School of Medicine, Pusan National University, Yangsan 50612, Republic of Korea; saylee@pnu.edu (S.Y.L.); younghye82@naver.com (Y.H.C.); everblue124@daum.net (E.J.P.); ylee23@gmail.com (Y.L.); s1jungin@hanmail.net (J.I.C.); sweetpea85@naver.com (S.R.L.); brain6@hanmail.net (R.J.K.); soo890624@naver.com (S.M.S.); 3 Department of Neurosurgery, Pusan National University Hospital, Busan 49241, Republic of Korea; spine@pusan.ac.kr; 4 Family Medicine Clinic, Obesity, Metabolism and Nutrition Center, Pusan National University Yangsan Hospital, Yangsan 50612, Republic of Korea; 5 Department of Family Medicine and Biomedical Research Institute, Pusan National University Yangsan Hospital, Yangsan 50612, Republic of Korea

**Keywords:** type 2 diabetes mellitus, cancer, DPP4 inhibitors, metformin

## Abstract

**Simple Summary:**

Type 2 diabetes mellitus (T2DM) is known to be related to an increased risk of several cancers. However, the effects of specific glucose-lowering drugs on cancer development remain uncertain. In this large multicenter cohort study using databases from 11 hospitals in Korea, we compared patients prescribed dipeptidyl peptidase-4 inhibitors (DPP4is) and/or metformin with those treated with other glucose-lowering drugs. After carefully balancing the groups, our findings showed that the group treated with DPP4is and/or metformin had a significantly lower risk of cancer, with consistent results across all institutions. These results suggest that metformin and DPP4is may have a protective role against cancer in T2DM patients, supporting their safety and potential benefits for long-term health outcomes.

**Abstract:**

Background/Objectives. Type 2 diabetes mellitus (T2DM) has been linked to an increased risk of several cancers. However, the influence of metformin and dipeptidyl peptidase-4 inhibitors (DPP4is) on the risk of cancers remains unclear. We investigated the association between using DPP4is and/or metformin and cancer risk compared with other glucose-lowering drugs (GLDs). Methods. This retrospective multicenter cohort study was performed using 11 hospital databases standardized to the OMOP Common Data Model (CDM) within the Observational Health Data Sciences and Informatics (OHDSI) network. T2DM patients using only DPP4is and/or metformin (DPP4is/Met group) were compared with those using other GLDs (other GLD group). From 413,344 eligible patients, propensity score (PS) 1:1 matching yielded 6674 patients in each group. Cox proportional hazards models were used to analyze cancer risk, and a random-effects meta-analysis was performed to calculate hazard ratios (HRs). Results. The DPP4is/Met group exhibited a significantly lower risk of incident cancer than the other GLD group (HR, 0.54; 95% CI, 0.41–0.69). This association was consistent across all hospitals. Regarding cancer-specific distributions, the DPP4is/Met group showed lower proportions of breast and prostate cancers, whereas the other GLD group showed higher proportions of lower gastrointestinal cancers. Conclusions. In this large multicenter study, using DPP4is and metformin showed a substantial association with a lower risk of cancer in T2DM patients relative to other GLDs. These findings suggest a potential protective effect of metformin and support the neutral-to-beneficial effect on cancer of DPP4is.

## 1. Introduction

Type 2 diabetes mellitus (T2DM) is known to be associated with an increased risk of several cancers, including pancreatic, liver, breast, and colorectal malignancies [1,2]. This association is thought to be mediated by mechanisms such as hyperinsulinemia, chronic inflammation, and metabolic dysregulation [3]. Given these overlapping pathways, glucose-lowering therapies have been investigated for their potential to alter cancer risk [4].

Among glucose-lowering agents, metformin has been the most extensively investigated in relation to cancer risk. Previous studies have suggested that metformin may reduce cancer incidence and mortality, including breast and colorectal cancers [5,6]. However, findings have been inconsistent, and several large-scale analyses did not demonstrate any association [7,8]. Sulfonylureas and insulin have been linked to higher risks of certain cancers, particularly pancreatic cancer [9]. Evidence regarding dipeptidyl peptidase-4 inhibitors (DPP4is) remains conflicting, with some studies reporting potential increases in pancreatic or thyroid cancer risk, while others found no significant associations [10,11].

Although considerable study has been conducted, most prior studies have been conducted within single centers or heterogeneous datasets, limiting comparability and generalizability. To overcome this limitation, large-scale multicenter studies using a standardized data structure and analytic framework are required.

Therefore, the purpose of this study was to examine the association between the use of DPP4is and/or metformin and the risk of incident cancer in comparison to other glucose-lowering drugs in patients with T2DM. To achieve this, we performed a retrospective multicenter cohort study at 11 hospitals in Korea using the Observational Health Data Sciences and Informatics (OHDSI) network and the Observational Medical Outcomes Partnership (OMOP) Common Data Model (CDM) [12,13].

## 2. Materials and Methods

### 2.1. Network and Tools

A multicenter retrospective cohort study was conducted inside the OHDSI collaborative’s distributed research network, using the OMOP CDM [12,13]. Each participating hospital transformed its electronic health records into the OMOP CDM format, which allowed for standardized analysis across institutions.

This study used data from eleven hospital databases to investigate differences in the risk of cancer between the target and comparator cohorts. To assess and correct for remaining unmeasured confounding, we employed methods like 1:1 propensity score (PS) matching, and negative controls available in the ATLAS tool (Copyright © 2025 Observational Health Data Sciences and Informatics; https://ohdsi.org/analytic-tools/, accessed on 11 March 2025) [14]. Cohort definitions as well as statistical analyses were executed through the OHDSI ATLAS platform and custom R packages (4.5.1).

### 2.2. Data Source and Study Population

This study is a retrospective multicenter cohort study using electronic health record data from 11 hospitals in Korea, which are part of the OHDSI community. At Pusan National University Hospital (PNUH), analyses were conducted using a single-hospital OMOP CDM to include the most recent data, whereas the remaining 10 institutions utilized OMOP CDMs within a common distributed research network. The participating institutions included Ajou University Hospital (AJUH, 3.0 million [M]; January 1994–March 2025), Daegu Catholic University Hospital (DCMC, 0.9 M; January 2005–March 2025), Gyeongsang National University Hospital (GNUH, 0.7 M; October 2009–March 2025), Kyung Hee University Medical Center (KHUM, 1.2 M; January 2008–March 2025), Keimyung University Dongsan Hospital (KUDH, 0.5 M; November 2018–March 2025), Myongji Hospital (MJH, 1.0 M; September 2003–March 2025), Pusan National University Hospital (PNUH, 1.0 M; January 2011–December 2023), Soonchunhyang University Hospital Bucheon (SCHBUH, 1.4 M; February 2001–April 2023), Soonchunhyang University Hospital Cheonan (SCHCAH, 1.1 M; May 2003–April 2023), Soonchunhyang University Hospital Gumi (SCHGUH, 0.8 M; August 2003–April 2023), and Soonchunhyang University Hospital Seoul (SCHSUH, 1.2 M; January 2001–April 2023). All data originated from claims records. To maximize data utilization, we employed variable observation periods for each hospital.

All databases were mapped to OMOP CDM version 5.3.0. This allowed common analysis codes to be shared across the research network, as the consistent healthcare data format and the standardized disease coding systems were established. In the OHDSI network framework, all statistical analyses and access to anonymous patient data took place within each institution’s firewall. Accordingly, only aggregate results without individual-level information were collected. This study was approved by the Institutional Review Board of Pusan National University Hospital (approval ID: 2412-004-145). The requirement for informed consent was exempted because of its retrospective design and the use of anonymized data.

**Figure 1:**
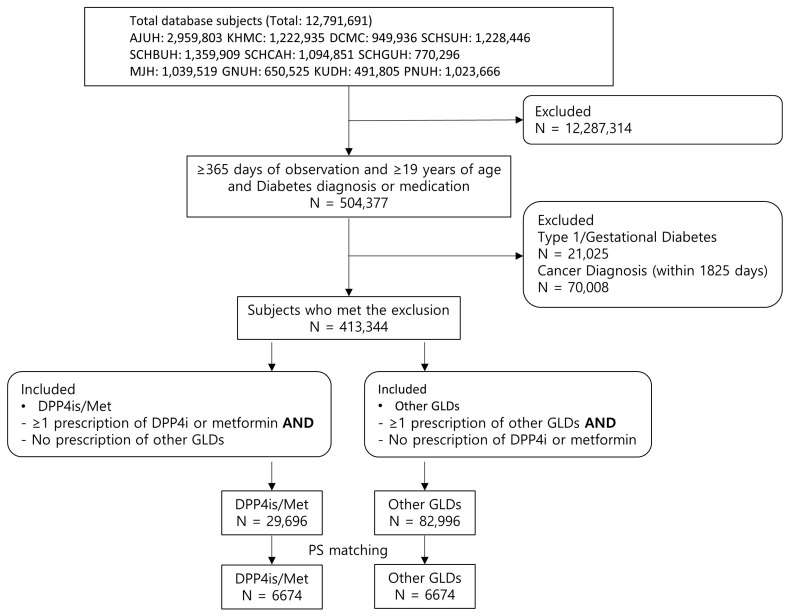
Flow chart for cohort selection process for the multicenter study.

The total database subjects across 11 hospitals were 12,791,691. The study population included adult patients (≥19 years) who were diagnosed with diabetes or prescribed glucose-lowering drugs prior to 26 March 2025, and had at least 365 days of continuous observation before the index date. We excluded patients who were diagnosed with type 1 diabetes mellitus or gestational diabetes within 180 days before or at any time after the index event and with a history of cancer within five years prior to the index date. A total of 413,344 eligible patients remained and were subsequently classified into two groups. The DPP4is/Met group was defined as those prescribed DPP4is and/or metformin at least once during the entire observation period, with no exposure to any other glucose-lowering drugs. The other GLD group was defined as those with neither record of DPP4is nor metformin use and at least one prescription of other glucose-lowering drugs. In both groups, only the first eligible event per individual was included (Figure 1). Cohort exit was defined as the discontinuation of the respective drug exposure, allowing gaps of up to 30 days to be considered continuous prescriptions. The end of follow-up was set at seven days after the last prescription for both groups.

### 2.3. Exposure

The date of the first prescription of the target medication (DPP4is and/or metformin for the DPP4is/Met group, other GLDs for the other GLD group) was set as the index date. Continuous exposure was defined by allowing less than a 30-day gap between prescriptions. The follow-up period for patients was defined as the duration from the index date to cancer occurrence, death, or the end of the observation period.

We used OHDSI’s large population to compare the proportions of medication use in the DPP4is/Met group and the other GLD group across hospitals. The list includes the following therapeutic classes: **DPP4is** (alogliptin [43013884], anagliptin [43008991], evogliptin [43009051], gemigliptin [43009089], linagliptin [40239216], sitagliptin [1580747], saxagliptin [40166035], teneligliptin [43009070], and vildagliptin [19122137]), **Metformin** [1503297]), and **various types of insulins** (regular human [1596977], glargine [1502905], lispro [1550023], degludec [35602717], aspart human [1567198], glulisine human [544838], and detemir [1516976]). **Other classes included sulfonylureas** (glibenclamide [43239982], gliclazide [19059796], glimepiride [1597756]), meglitinides (mitiglinide [43009094], nateglinide [502826], repaglinide [1516766]), thiazolidinediones (pioglitazone [1525215], lobeglitazone [43009055]), **SGLT2 inhibitors** (dapagliflozin [44785829], empagliflozin [45774751], ertugliflozin [793293], ipragliflozin [43009020]), **GLP-1 receptor agonists** (dulaglutide [45774435], exenatide [1583722], liraglutide [40170911], lixisenatide [44506754], semaglutide [793143]), and **alpha-glucosidase inhibitors** (acarbose [1529331], voglibose [43009032]).

### 2.4. Outcomes

The primary endpoint was the new occurrence of any cancer after the index date. Cancer was defined using 477 standardized condition concept IDs within the OMOP CDM vocabulary system. Events were identified at the first incident cancer diagnosis during follow-up, and person-time was calculated to estimate incidence rates, with a 90-day time-at-risk window applied after cohort entry.

### 2.5. Statistical Analysis

Baseline characteristics (age, sex, Charlson comorbidity index) were compared between the two groups in each hospital before and after propensity score (PS) adjustment. PS was estimated using large-scale logistic regression with L1-regularization and included demographics, comorbidities, and concomitant medications. A 1:1 PS matching was applied. Covariate balance was assessed by standardized differences, with an absolute value < 0.1 considered well balanced.

Incidence rates (IRs) per 1000 person-years (PY) and hazard ratios (HRs) with 95% confidence intervals (CIs) were calculated by each hospital using Cox proportional hazards models. Minimum detectable relative risks (MDRRs) were also estimated to assess statistical power within each institution. A random-effects model in R software (version 4.2.1; R Foundation for Statistical Computing, Vienna, Austria) was used for meta-analysis, and heterogeneity across institutions was quantified using the I^2^ statistic.

A *p*-value < 0.05 was considered statistically significant. This study used OHDSI’s ATLAS tool to analyze data, version 2.7.6 for the ten participating hospitals and version 2.13.0 for PNUH (accessed on 11 March 2025; Copyright © 2025 Observational Health Data Sciences and Informatics: https://ohdsi.org/analytic-tools/). The analytic code can be executed in any database structured according to the OMOP CDM, allowing replication of the analyses using the same methods.

## 3. Results

Across all databases, we extracted 29,696 T2DM patients with DPP4is and/or metformin (DPP4is/Met group) and 82,996 T2DM patients with other GLDs (other GLD group). After 1:1 matching, 6674 patients of DPP4is/Met and other GLD groups were finally selected (Figure 1). After PS adjustment, age, gender, and Charlson index did not significantly differ between the DPP4is/Met and other GLD groups across institutions, except for age at MJH (standardized difference [SD], 0.640), gender at SCHGUH (SD, 0.135), and Charlson index at GNUH (SD, 0.152) and KUDH (SD, –0.144) (Table 1).

Across participating hospitals, the incidence rates (IRs) of any cancer were generally lower in the DPP4is/Met group compared with the other GLD group (IRs per 1000 person-years; 9.79 vs. 15.09 at AJUH, 7.04 vs. 23.98 at KHMC, and 20.62 vs. 44.37 at PNUH, etc.) (Table 2). However, the minimum detectable relative risk (MDRR) ranged from 1.98 at AJUH to over 4.7 at MJH, GNUH, and SCHGUH, suggesting insufficient statistical power in some institutions. Overall, while most hospitals showed lower event rates in the DPP4is/Met group, results from smaller institutions with limited sample sizes should be interpreted with caution due to wide MDRR values.

**Figure 2:**
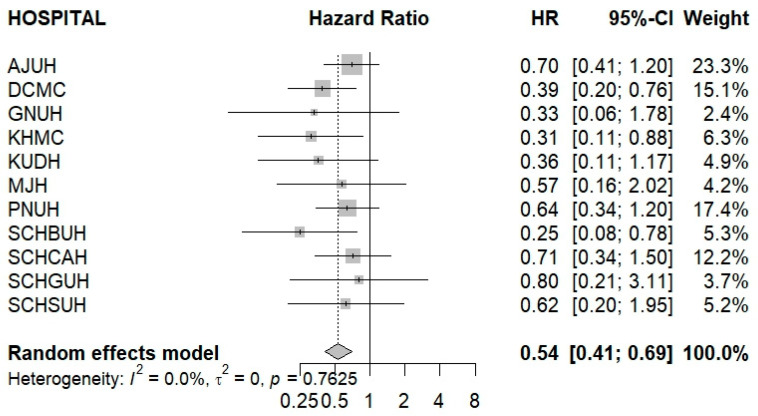
Forest plot of meta-analysis evaluating cancer risk in the DPP4is/Met group compared with the other GLD group. Abbreviations: AJUH, Ajou University Hospital; DCMC, Daegu Catholic University; GNUH, Gyeongsang National University Hospital; KHMC, Kyung Hee University Medical Center; KUDH, Keimyung University Dongsan Hospital; MJH, Myongji Hospital; PNUH, Pusan National University Hospital; SCHBUH, Soonchunhyang University Hospital Bucheon; SCHCAH, Soonchunhyang University Hospital Cheonan; SCHGUH, Soonchunhyang University Hospital Gumi; SCHSUH, Soonchunhyang University Hospital Seoul; HR, Hazard ratio; CI, Confidence interval.

The DPP4is/Met group was associated with a significantly lower risk of any cancer compared with the other GLD group (HR, 0.54; 95% CI, 0.41–0.69). Although confidence intervals were wide for some institutions due to limited sample sizes, none of the estimates indicated an increased risk in the DPP4is/Met group. The direction of effect was consistent across hospitals, and no heterogeneity was observed (I^2^ = 0.0%, *p* = 0.76) (Figure 2).

**Figure 3:**
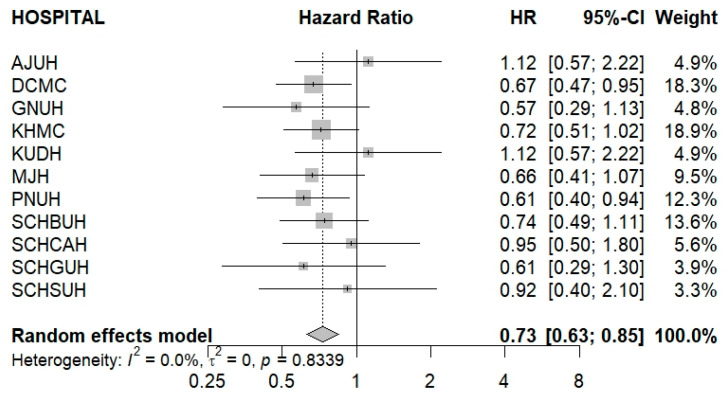
Forest plot of meta-analysis evaluating cancer risk in the DPP4is/Met group compared with the other GLD group in Patients with ≥180 Days of Drug Exposure. Abbreviations: AJUH, Ajou University Hospital; DCMC, Daegu Catholic University; GNUH, Gyeongsang National University Hospital; KHMC, Kyung Hee University Medical Center; KUDH, Keimyung University Dongsan Hospital; MJH, Myongji Hospital; PNUH, Pusan National University Hospital; SCHBUH, Soonchunhyang University Hospital Bucheon; SCHCAH, Soonchunhyang University Hospital Cheonan; SCHGUH, Soonchunhyang University Hospital Gumi; SCHSUH, Soonchunhyang University Hospital Seoul; HR, Hazard ratio; CI, Confidence interval.

The DPP4is/Met group was associated with a statistically significantly lower risk of any cancer compared with the other GLD group when considering patients with 180 days or more of drug exposure (Figure 3). The pooled Hazard Ratio was 0.73 (95% CI, 0.63–0.85), indicating a 27% reduction in cancer risk in the DPP4is/Met group. While most institutions showed a trend towards risk reduction (HR < 1.00), the result from DCMC (HR, 0.67; 95% CI, 0.47–0.95) was individually significant. Importantly, the analysis revealed no significant heterogeneity among institutions (I^2^ = 0.0%, *p* = 0.8339), suggesting a consistent protective effect of DPP4is/Met in the long term.

**Figure 4:**
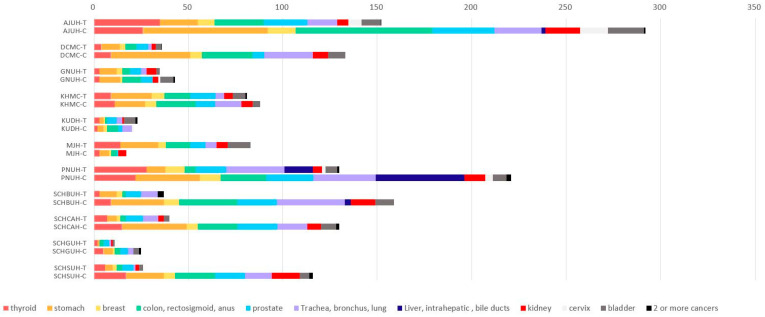
Distribution of Specific Cancer Types in the DPP4is/Met group (T) vs. the other GLD group (C) Across Multiple OMOP CDM Databases. This stacked bar chart presents the number of major cancer cases categorized by specific cancer type, comparing the use of DPP4is/Met group with the other GLD group across multiple clinical institutions. The counts reflect observed cases of selected major cancer types only and do not represent total cancer incidence or statistical significance. Abbreviations: AJUH, Ajou University Hospital; DCMC, Daegu Catholic University; GNUH, Gyeongsang National University Hospital; KHMC, Kyung Hee University Medical Center; KUDH, Keimyung University Dongsan Hospital; MJH, Myongji Hospital; PNUH, Pusan National University Hospital; SCHBUH, Soonchunhyang University Hospital Bucheon; SCHCAH, Soonchunhyang University Hospital Cheonan; SCHGUH, Soonchunhyang University Hospital Gumi; SCHSUH, Soonchunhyang University Hospital Seoul.

Figure 4 shows the distribution and observed count of major cancer cases across participating clinical institutions, comparing the DPP4is/Met group (denoted by T) with the other GLD group (denoted by C). This analysis is descriptive in nature, and no statistical testing was performed. Overall, the number of cancer cases varied substantially by institution and treatment group. The AIUH-C group exhibited the highest total count, whereas KUH showed the lowest across both groups. In most institutions, including AIUH, DCMC, and PNUH, the C group demonstrated equal or higher counts of major cancers compared with the T group. Institution-specific patterns were also observed: at AIUH, the C group had more liver and bile duct cancers, whereas the T group showed higher counts of colon and rectal cancers; at PNUH, the C group showed more liver and bile duct cancers along with several bladder cancer cases; and at SCHBUH and SCHCAH, the C groups exhibited higher proportions of trachea, bronchus, and lung cancers. Common cancers, including thyroid, breast, and prostate, were consistently observed in both groups across institutions.

**Figure 5:**
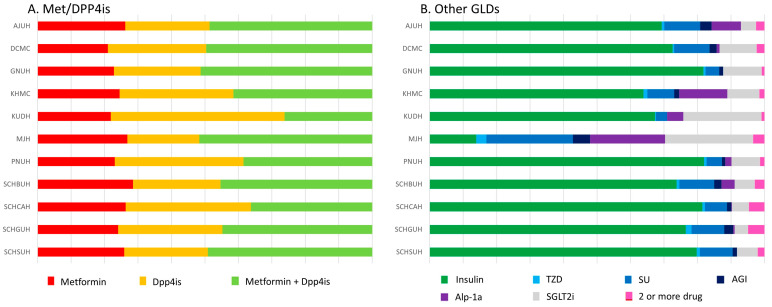
Distribution of GLD use in the DPP4is/Met group vs. the other GLD group Across Multiple OMOP CDM Databases. Abbreviations: AJUH, Ajou University Hospital; DCMC, Daegu Catholic University; GNUH, Gyeongsang National University Hospital; KHMC, Kyung Hee University Medical Center; KUDH, Keimyung University Dongsan Hospital; MJH, Myongji Hospital; PNUH, Pusan National University Hospital; SCHBUH, Soonchunhyang University Hospital Bucheon; SCHCAH, Soonchunhyang University Hospital Cheonan; SCHGUH, Soonchunhyang University Hospital Gumi; SCHSUH, Soonchunhyang University Hospital Seoul.

The analysis of prescription patterns is presented in Figure 5. In most institutions, the DPP4is/Met group (A) was predominantly treated with a combination of DPP4is and metformin. The other GLD group (B) demonstrated a more complex and diverse treatment regimen, including insulin, SUs, and TZDs, with insulin use being predominant across the majority of hospitals. Although the exact proportions varied by institution, the overall prescribing patterns were generally similar.

## 4. Discussion

The purpose of this study was to assess the association between the use of DPP4is and/or metformin and a lower risk of incident cancer compared with other glucose-lowering drugs (GLDs) in type 2 diabetes mellitus (T2DM) patients. Using data from 11 hospitals mapped to the OMOP-CDM within the OHDSI research network, we found that using DPP4is and/or metformin was linked to a substantial reduction in the risk of overall cancer (HR, 0.54; 95% CI, 0.41–0.69). This protective association was consistent across hospitals, and no heterogeneity was observed.

The potential impact of DPP4is on cancer risk remains a subject of debate. Earlier studies raised concerns regarding possible associations with pancreatic and thyroid cancers [15]. However, accumulating evidence from large-scale randomized controlled trials and meta-analyses has not substantiated these findings, indicating that DPP4is use is not associated with an increased incidence of malignancy [16,17]. Indeed, comprehensive analyses have shown no significant association between DPP4is use and pancreatic cancer, though a modest risk of acute pancreatitis cannot be entirely excluded [18]. Beyond their established safety, previous studies suggest that DPP4is may exert protective effects against certain cancers [19]. A meta-analysis reported reduced incidences of rectal and skin cancers among patients treated with DPP4is [20], and preclinical studies indicate potential immunomodulatory and antifibrotic mechanisms that could contribute to anti-tumor activity [21]. Observational analyses have also reported improved survival in colorectal and lung cancer patients treated with DPP4is [22], supporting the hypothesis of pharmacological effects beyond glucose lowering.

Metformin has been more consistently associated with protective effects against cancer. Its proposed mechanisms include enhanced insulin sensitivity, reduced circulating insulin and IGF-1 levels, and activation of the AMPK–mTOR signaling pathway, collectively suppressing oncogenic signaling and tumor proliferation. Studies further demonstrated that metformin can trigger cell cycle arrest, stimulate apoptosis, and suppress tumor metabolism, including reversal of the Warburg effect [23]. Several studies have aligned with these mechanistic insights, with case–control and cohort studies reporting reductions in overall cancer risk among metformin users, as well as decreased incidence of gastrointestinal cancers and improved outcomes in breast and colorectal cancers [24,25].

In this study, the comparator group (other GLD group) was predominantly treated with insulin and sulfonylureas (SUs) across most institutions. While sodium–glucose cotransporter-2 inhibitors (SGLT2 inhibitors) have been associated with reduced cancer risk [26,27], insulin and SUs have been linked to increased risks of overall, pancreatic, and liver cancers [28,29]. The lower cancer incidence observed in the DPP4is/Met group is likely attributed to these prescribing patterns.

Overall, our multicenter cohort analysis demonstrated that using DPP4is and/or metformin was not linked to an increased risk of cancer but was instead linked to an overall reduction in cancer incidence. These results align with prior systematic reviews and meta-analyses, supporting the evidence that DPP4is and metformin are not related to risk of cancer and may confer protective effects against certain malignancies.

A favorable outcome from our study is the lower cancer incidence observed in the DPP4is/Met group. While this difference might be attributable to a direct anti-cancer effect of the medication, it’s also plausible that confounding factors, such as the “Healthy User Effect,” have played a role. This effect is linked to the tendency for DPP4is/Met to be prescribed to patients in the early stages of diabetes. These individuals may be relatively younger and have fewer comorbidities compared to patients using other antidiabetic medications, such as insulin. Consequently, their lower cancer incidence might be a reflection of their healthier baseline status, not solely the drug’s influence.

Our preliminary analysis was based on a binary user-group comparison. However, to more rigorously assess the association, a more granular analysis is required to understand the impact of cumulative drug exposure. We will therefore compare the cumulative dose and duration of use for both the DPP4is/Met and other GLD cohorts. This will help to elucidate whether the observed lower cancer incidence is a direct consequence of long-term drug use or merely a reflection of a patient population with a shorter median duration of exposure, thereby reducing their overall time at risk.

Site-specific analysis of cancer proportions further supports the hypothesis that the benefits of DPP4is and metformin may be more pronounced for certain malignancies, such as breast, prostate, stomach, and colorectal cancers. This observation aligns with existing literature on the potential chemopreventive effects of DPP4is and metformin on various cancer types [30]. However, it is crucial to recognize that this data reflects the proportional distribution of cancer types within each cohort, not the absolute incidence rates. Consequently, these findings do not eliminate the influence of confounding factors, such as the “Healthy User Effect”, which may indicate that the patients prescribed DPP4is/Met had a lower baseline risk for these specific cancers to begin with.

This analysis does not establish a direct causal relationship, but its comprehensive design enabled a more nuanced understanding of the cohorts. The detailed evaluation of prescription patterns and site-specific cancer distributions allowed us to discuss the significant influence of patient characteristics and the “Healthy User Effect.” In addition, these findings offer a strong rationale for future, more focused investigations into the specific impact of DPP4is/Met therapy on cancer pathogenesis.

This study has several limitations. First, we were unable to analyze the difference in cancer risk among metformin monotherapy, DPP4is monotherapy, and their combined therapy, as the number of cancer cases in the DPP4is monotherapy group was very small. Consequently, it remains uncertain whether cancer risk varies among metformin use, DPP4is use, and their combined therapy, and additional studies are required to clarify the specific impact of each treatment strategy. Second, as an observational study, residual confounding by unmeasured variables such as smoking, alcohol consumption, diet, and physical activity cannot be excluded. Third, cancer diagnoses were based on administrative codes, raising the possibility of misclassification. Fourth, follow-up duration was relatively short in some institutions, as reflected in wide MDRRs, limiting evaluation of long-term outcomes. Finally, although CDM-based standardization reduced heterogeneity, inter-institutional differences in prescribing patterns and data quality may still have influenced results.

## 5. Conclusions

In conclusion, this large multicenter study demonstrated that metformin and DPP4is use were linked to a significantly reduced risk of incident cancer compared with other GLDs in patients with T2DM. These findings support the potential protective role of metformin and reinforce the neutral-to-beneficial cancer safety profile of DPP4is. Future research should investigate cancer risk across specific tumor types and drug combinations, while also accounting for potential confounding factors such as the “Healthy User Effect”.

## Figures and Tables

**Table 1:** Demographic factors of patients with T2DM treated with DPP4is and/or metformin versus other GLDs.

		Before PS Adjustment			After PS Adjustment		
		DPP4is/Met	Other GLDs	Std_Diff	DPP4is/Met	Other GLDs	Std_Diff
Age (years)	AJUH	59.7	58.4	−0.01	60.3	60.8	−0.005
	DCMC	64.3	63.9	−0.011	65.3	65.1	0.006
	GNUH	66.5	69.1	−0.009	61.1	61.8	−0.011
	KHMC	66.5	64.5	−0.036	66.1	66.7	<0.001
	KUDH	66.5	65.9	−0.014	66.9	66.7	−0.004
	MJH	70.2	65.7	−0.045	68.9	68.6	−0.003
	PNUH	65.3	66.1	−0.028	65.8	65.2	0.002
	SCHBUH	61.7	63.3	−0.034	62.5	62.3	−0.003
	SCHCAH	63.2	66.3	−0.034	65.8	64.9	−0.01
	SCHGUH	62.2	63.6	0.014	60.2	60.4	<0.001
	SCHSUH	62.5	63.2	0.002	63.4	63.0	−0.003
Male (%)	AJUH	60.4	53.0	0.15	56.3	56.2	0.003
	DCMC	54.4	53.3	0.022	52.3	53.1	−0.016
	GNUH	56.2	58.6	−0.048	61.0	54.8	0.126
	KHMC	47.8	45.8	0.041	45.7	43.9	0.036
	KUDH	51.9	51.1	0.016	55.8	54.2	0.03
	MJH	47.2	45.3	0.039	48.3	48.6	−0.005
	PNUH	48.7	54.4	−0.115	52.7	50.6	0.041
	SCHBUH	54.7	51.0	0.074	54.3	50.4	0.079
	SCHCAH	54.6	53.0	0.031	51.5	53.7	−0.044
	SCHGUH	54.9	55.5	−0.011	61.6	55.0	0.135
	SCHSUH	54.4	49.4	0.099	49.8	49.5	0.007
Charlson index	AJUH	1.801	1.674	0.086	1.801	1.836	−0.029
	DCMC	2.177	2.143	0.023	2.396	2.389	0.005
	GNUH	1.304	1.489	−0.132	2.224	2.046	0.152
	KHMC	2.216	2.138	0.049	2.494	2.505	−0.008
	KUDH	2.048	1.407	0.482	2.076	2.255	−0.144
	MJH	2.139	2.134	0.003	2.445	2.421	0.016
	PNUH *						
	SCHBUH	2.014	2.093	−0.051	2.009	2.052	−0.033
	SCHCAH	1.742	2.031	−0.18	1.926	1.901	0.019
	SCHGUH	1.837	1.814	0.015	1.815	1.848	−0.026
	SCHSUH	2.362	2.656	−0.173	2.595	2.608	−0.008

* At PNUH, analyses were conducted using a single-hospital OMOP CDM, where differences in the underlying coding system did not allow calculation of the Charlson index within ATLAS. Abbreviations: AJUH, Ajou University Hospital; DCMC, Daegu Catholic University; GNUH, Gyeongsang National University Hospita, KHMC, Kyung Hee University Medical Center; KUDH, Keimyung University Dongsan Hospital; MJH, Myongji Hospital; PNUH, Pusan National University Hospital; SCHBUH, Soonchunhyang University Hospital Bucheon, SCHCAH, Soonchunhyang University Hospital Cheonan; SCHGUH, Soonchunhyang University Hospital Gumi; SCHSUH, Soonchunhyang University Hospital Seoul; DPP4is, DPP4 inhibitors; GLDs, glucose-lowering drugs.

**Table 2:** The risk of cancer in T2DM patients prescribed with DPP4is and/or metformin compared with other GLDs.

	1:1 Matching								
	DPP4is/Met				Other GLDs				
	Patients	Person-Years	Events	IR	Patients	Person-Years	Events	IR	MDRR
AJUH	1339	2758	27	9.79	1339	2651	40	15.09	1.98
KHMC	788	1703	12	7.04	788	1626	39	23.98	2.19
DCMC	868	1913	14	7.32	868	1779	38	21.36	2.17
SCHSUH	592	1222	12	9.82	592	1131	14	12.37	3.00
SCHBUH	762	1607	7	4.35	762	1511	20	13.23	2.94
SCHCAH	635	1251	14	11.19	635	1172	22	18.76	2.54
SCHGUH	211	452	5	11.05	211	408	7	17.16	5.04
MJH	420	810	5	6.17	420	770	8	10.39	4.73
GNUH	241	488	5	10.23	241	474	8	16.86	4.73
KUDH	330	613	7	11.42	330	671	12	17.86	3.62
PNUH	488	1018	21	20.62	488	969	43	44.37	2.01

Abbreviations: AJUH, Ajou University Hospital; DCMC, Daegu Catholic University; GNUH, Gyeongsang National University Hospita, KHMC, Kyung Hee University Medical Center; KUDH, Keimyung University Dongsan Hospital; MJH, Myongji Hospital; PNUH, Pusan National University Hospital; SCHBUH, Soonchunhyang University Hospital Bucheon, SCHCAH, Soonchunhyang University Hospital Cheonan; SCHGUH, Soonchunhyang University Hospital Gumi; SCHSUH, Soonchunhyang University Hospital Seoul; IR, incidence rate per 1000 person-years; MDRR, Minimum detectable relative risks; DPP4is, DPP4 inhibitors; GLDs, glucose-lowering drugs.

## Data Availability

The data that support the findings of this study are available from the OHDSI study, but restrictions apply to their availability. These data were used under license for the current study, and are not publicly available. The outcome data and codes are, however, available from the authors upon reasonable request, and with permission of the OHDSI study.
